# Nutritional Approach to Prevention and Treatment of Cardiovascular Disease in Childhood

**DOI:** 10.3390/nu13072359

**Published:** 2021-07-10

**Authors:** Maria Elena Capra, Cristina Pederiva, Claudia Viggiano, Raffaella De Santis, Giuseppe Banderali, Giacomo Biasucci

**Affiliations:** 1 Centre for Paediatric Dyslipidaemias, Paediatrics and Neonatology Unit, Guglielmo da Saliceto Hospital, 29121 Piacenza, Italy; g.biasucci@ausl.pc.it; 2 Clinical Service for Dyslipidaemias, Study and Prevention of Atherosclerosis in Childhood, Pediatrics Unit, ASST-Santi Paolo e Carlo, 20142 Milan, Italy; cristina.pederiva@asst-santipaolocarlo.it (C.P.); claudia.viggiano@unimi.it (C.V.); raffaella.desantis@unimi.it (R.D.S.); giuseppe.banderali@asst-santipaolocarlo.it (G.B.)

**Keywords:** nutrition in childhood, primordial prevention, dietary treatment, hypercholesterolemia, CHD prevention

## Abstract

Coronary Heart Disease (CHD) is a major mortality and morbidity cause in adulthood worldwide. The atherosclerotic process starts even before birth, progresses through childhood and, if not stopped, eventually leads to CHD. Therefore, it is important to start prevention from the earliest stages of life. CHD prevention can be performed at different interventional stages: primordial prevention is aimed at preventing risk factors, primary prevention is aimed at early identification and treatment of risk factors, secondary prevention is aimed at reducing the risk of further events in those patients who have already experienced a CHD event. In this context, CHD risk stratification is of utmost importance, in order to tailor the preventive and therapeutic approach. Nutritional intervention is the milestone treatment in pediatric patients at increased CHD risk. According to the Developmental Origin of Health and Disease theory, the origins of lifestyle-related disease is formed in the so called “first thousand days” from conception, when an insult, either positive or negative, can cause life-lasting consequences. Nutrition is a positive epigenetic factor: an adequate nutritional intervention in a developmental critical period can change the outcome from childhood into adulthood.

## 1. Introduction

### 1.1. Coronary Heart Disease and Atherosclerosis

Coronary Heart Disease (CHD) is one of the main causes of death in adulthood in the United States and in Western countries [1,2]. CHD mainly causes morbidity and mortality in adult subjects, but lipid deposition and arterial damage dates back to the early stages of life [3,4]. The atherosclerotic process starts even before birth; post-mortem studies showed that fatty-streaks are already detectable in human fetal aortas, greatly increased by maternal hypercholesterolemia [3,4]. The prolonged and sustained arterial exposure to elevated low-density lipoprotein cholesterol (LDL-C) increases cholesterol deposition and enhances the atherosclerotic process, eventually leading to CHD [5]. The exposure to factors associated with increased cardiovascular risk exacerbates the atherosclerotic cascade [6]. Atherosclerosis is already present in childhood, for this reason prevention of atherosclerosis related diseases should be present and mandatory from the earliest stages of life. Health professionals dealing with children and adolescents are the main actors of this preventive battle, and the knowledge, the detection and the treatment of CHD risk are their cornerstone swords. In this narrative review we focus on nutritional intervention in children and adolescents at high cardiovascular risk, starting from the historical data of the literature up to the latest indications of the most recent guidelines. In childhood, cardiovascular risk has been often referred to a single disease or condition, whereas in our review we have tried to consider cardiovascular risk and nutritional intervention as the “trait d’union” of a variety of conditions present in childhood and adolescence. We have looked at childhood and adolescence focusing on cardiovascular-disease risk factors, so as to spot and unify all the main conditions at high CHD risk in these age groups. In the near future, further studies will be needed to sharpen even more this approach, thus making nutritional intervention more tailored and effective.

### 1.2. Paediatric Patients and Cardiovascular Risk

CHD prevention can be performed at different stages of intervention: primordial prevention is aimed at preventing risk factors, primary prevention is aimed at early identification and treatment of risk factors, whereas secondary prevention is aimed at reducing the risk of further events in those patients who have already experienced a CHD event. CHD risk factors can be present since the early stages of life, both on a genetic and on a metabolic basis. The presence of classical severe CHD risk factors and the presence of heart diseases, that make the child more vulnerable to CHD, are both underlying conditions for increased CHD in childhood and/or in the following stages of life. Detection of CHD risk factors and risk stratification is an issue of utmost importance in the management of paediatric patients with cardiovascular risk. In this context, the first step is the knowledge of the conditions that enhance CHD risk in childhood, their detection and their adequate treatment [7]. The extent of the risk for atherosclerotic coronary artery disease, compared to the general population, has been proposed as a model for CHD risk stratification [8]. Children and adolescents with underlying heart diseases are at increased CHD risk as well. In particular, subjects with coronary artery anomalies must be strictly supervised. On a pathogenetic basis, three underlying conditions seem to increase CHD risk: inflammation, insulin resistance and oxidative stress [9]. Inflammation seems to be the main causative condition of CHD risk through elevated triglycerides levels, low high-density lipoprotein (HDL) cholesterol (HDL-C) levels and increase in small LDL-C particles. Inflammation causes oxidative stress and requires an increased glucose intake, thus triggering insulin resistance. Congenital or acquired diseases and conditions can be classified as “high risk”, “moderate risk” or “at risk” for CHD, according to the extent of the coronary artery pathology compared to that of healthy population. Conditions that make the subject at high CHD risk include homozygous familial hypercholesterolemia (HoFH), diabetes mellitus type 1 and 2, end stage renal disease, Kawasaki disease with persistent aneurysms, childhood cancer survival. Conditions that cause a moderate CHD risk include heterozygous familial hypercholesterolemia (FH), severe obesity, hypertension, elevated blood level of lipoprotein(a). Diseases that make the affected subject at risk for CHD include obesity, chronic inflammatory diseases, Kawasaki disease with regressed aneurysms and some congenital heart defects. Once the CHD risk stratification has been carried out, it is important to assess the presence of other CHD risk factors and/or comorbidities, such as altered fasting lipid profile, smoking, positive family history for premature CHD in first-degree relatives, elevated blood pressure, overweight or obesity, altered fasting blood glucose, lack of physical activity. The extent and the strictness of intervention, both on a lifestyle and on a pharmacological basis, are determined by the risk stratification and the presence or absence of comorbidities [7,8].

### 1.3. Classical CHD Risk Factors

Among the classical CHD risk factors, FH is one of the most important and most frequent conditions. FH is a primitive dyslipidemia and is present in 1 per 200–250 subjects in the heterozygous form in the general population. Patients with FH are at increased risk of CHD disease because they have genetic conditions (mutations on LDL-Receptor gene, Apolipoprotein B gene, PCSK9 gene) that result in high LDL-C levels and can cause premature atherosclerosis; they frequently have elevated non-HDL-C levels (expression of highly atherogenic VLDL particles) and apoB/apoA ratio >1, recently recognized as a new risk factor for atherosclerosis [10]. If undetected and untreated, subjects with FH have increased mortality and morbidity. Early detection and treatment of FH helps “gaining decades of life”, as stated in the recent European Atherosclerosis Society Consensus [11]. Phenotypic expression of FH typically occurs in adulthood, so the detection of affected children and adolescents must be based on screening strategies. Unfortunately, knowledge of the “cholesterol problem” is still really poor in the general population, especially among young adults [12]. Lipoprotein(a) is a strong, genetically defined CHD risk factor. Lipoprotein(a) levels are stable from early childhood and high Lipoprotein(a) levels can help to identify FH patients at higher CHD risk [13,14]. Moreover, elevated Lipoprotein(a) levels have been linked to thromboembolic events in children and adolescents [15]. Childhood obesity is defined as body mass index (BMI) higher than 95th centile for age and sex. Obesity is a metabolic and public health emergency in the Western World starting from childhood and involving, in some countries, up to one third of 6–9 years old children [16]. Numerous studies [17,18,19] have correlated obesity with fatty streaks, atherosclerotic lesions and CHD morbidity and mortality. The American Academy of Pediatrics guidelines indicate children with obesity as children with “independent CHD risk factors” [20]. Severe obesity is defined as BMI higher than 35 kg/m^2^ and/or higher than 120% of the 95th centile for sex and age. Children and adolescents with severe obesity are a cluster at extremely elevated CHD risk, as they have subclinical atherosclerosis and endothelial activation [21,22]. Diabetes mellitus, both type 1 and type 2, is related to increased CHD risk. Subclinical atherosclerosis, shown as an increase in carotid intima media thickness, and microvascular damage have been demonstrated in children with diabetes mellitus [23]. Hyperglycemia is the primary mediator of atherosclerosis in subjects with diabetes mellitus, therefore the achievement of optimal glucose control is the first step in CHD risk reduction [24]. However, the maintenance of a balanced diet is of utmost importance for these subjects, as they often tend to assume an excessive intake of lipids and proteins. Hypertension is another risk factor for CHD [25], both isolated or associated to other diseases. Elevated blood pressure has been linked to higher level of carotid intima media thickness and to arterial damage [26].

### 1.4. High Risk Medical Conditions and Heart Disease

Chronic kidney disease is a vasculopathic state characterized by early abnormalities of vascular and cardiac function [27]. Numerous chronic inflammatory diseases, such as rheumatoid arthritis, inflammatory bowel disease and systemic lupus erythematosus, are characterized by an increased CHD risk in adulthood. Childhood cancer survivors represent another CHD high risk category. Several studies [28] have shown that childhood cancer survivors have greater insulin resistance and increased arterial stiffness compared with their healthy siblings, and this difference is maintained throughout adulthood. The pathogenesis of this increased cardiovascular risk is related to the vulnerability of these patients and it has a multifactorial basis. Moreover, anthracycline treatment has been linked to dilated cardiomyopathy [29]. Children with congenital heart disease are considered more vulnerable. The prevalence of congenital heart disease is approximately 9 per 1000 live births. The risk of CHD in subjects with congenital heart disease is related to the presence of obstructive lesions of the left ventricle and aorta, cyanotic congenital heart defects and coronary artery abnormalities [30]. An important cause of secondary heart disease is Kawasaki disease, a multisystemic vasculitis that can cause coronary artery aneurysms [31].

## 2. Nutritional Treatment

Nutritional treatment is the milestone intervention in pediatric patients at increased CHD risk. According to the Developmental Origin of Health and Disease theory, also known as “Barker hypothesis”, the origin of lifestyle-related disease begins with conception and proceeds through embryonic, fetal and neonatal stages by the relation between genes and the environment. In the so called “first thousand days” from conception, an insult, either positive or negative, can cause life-lasting consequences [32]. Barker discovered that ischemic heart disease incidence in adulthood is linked to low birth weight and malnutrition in the earliest stages of life [33]. According to Barker’s hypothesis, an individual is programmed towards nutritional thrift during gestation and early postnatal life so as he can survive environmental insults caused by poor nutrition [34]. More recent studies have shown that maternal malnutrition, either qualitative or quantitative, is responsible for shaping future offspring health; in particular, this refers to a maternal low protein and high fat diet [35,36]. According to the developmental programming by early life exposures, children born extremely preterm have higher systolic and diastolic blood pressures already at 2–3 and 6.5 years of age and preterm birth has been linked with higher blood pressure later in life [37,38]; finally, as recently reported, they have an increased risk for ongoing residual kidney injury and chronic kidney disease [39]. In this context, nutrition is a positive epigenetic factor: an adequate nutritional intervention in a developmental critical period can change the outcome in adulthood. This concept has a revolutionary power and supports the idea that nutritional treatment should be considered comparable to drug therapy.

### 2.1. Scientific Support for Dietary Recommendations

Nutritional intake of saturated fatty acids, trans fatty acids and cholesterol are widely known as main determinants of the increase of cholesterol blood levels and, subsequently, of the development of cardiovascular disease. Other cardiovascular risk factors are implied in this event as well. In the occurrence of many risk factors in one individual, the evidence of atherosclerotic lesions in the aorta and coronary arteries starting from in early childhood becomes more likely [4,17]. Longitudinal studies confirm that there is a tracking of overweight, hypercholesterolemia and hypertension from the earliest years of life into adulthood and that lifestyle and habits as unhealthy diet, excessive caloric intake, lack of physical activity and cigarette smoking play an important role in influencing the above-mentioned risk factors [26]. Intervention studies, aimed at measuring the efficacy and safety of diets poor in total and saturated fat and cholesterol, added further evidence. The meta-analysis of studies in adults suggested that introduction of low saturated-fat and low cholesterol diet lowers blood total and LDL-C levels. Pediatric studies confirmed the safety and efficacy of a low-cholesterol and low-saturated fat diet in children and adolescents. Among these, the Dietary Intervention Study in Children (DISC) was a randomized trial consisting in administration of a low-saturated fat and cholesterol diet for a 3 years period in American children aged 8 to 11 years; the intervention group received a diet with 28% of calories from total fat, 10% of calories from saturated fat and 95 mg per day of cholesterol, while the control group consumed 33 to 34% of calories as total fat, 12.7% of calories as saturated fat and 112 mg per day of cholesterol. No differences in anthropometric parameters nor in serum ferritin levels were found in the two groups; the intervention group showed lower levels of LDL-C and maintained psychological well-being [40]. Likewise, in the Special Turku Coronary Risk Factor Intervention Project for Babies (STRIP) randomized and prospective study, a low-saturated fat and low-cholesterol diet was introduced at a very young age (during weaning, at 7 months of life), with dietary education continued until the age of 20 years [41,42]. As for the intervention group, both studies used diets tailored according to current recommendations for therapeutic lifestyle changes to lower cholesterol levels, with total fat <30% of total calories and cholesterol intake <200 mg per day. Saturated fat intake, although not <7% of total calories, was significantly less than in children assigned to usual care. Non-adverse effects of the recommended intervention diets were observed in growth, neurological development, metabolic function and nutrient adequacy [43,44,45]. In both studies LDL-C levels were significantly lower among children in the intervention group compared with controls, and children who received nutritional intervention had higher chances of choosing healthy food [46].

### 2.2. Low Fat Diet in Children

According to Barker et al. [34], granting an adequate growth from birth and throughout childhood may be a fundamental milestone in the prevention of atherosclerosis, as the incidence of CHD is higher especially in males with low birth weight or low weight at 1 year of life. We have also learned that even a little reduction of the mean total and LDL-C values in childhood and adolescence could decrease substantially the incidence of CHD, when prolonged into adult life. It is a general thought that relative fat intake is high (35–55% of energy) during the first year of life, then decreasing toward adult values (30–35% of energy) in the following years. Fat is one of the main sources of energy, mainly in childhood, when growth is remarkably fast. In the first period of life, a great amount of daily energy is used for growth, therefore a high fat intake (40–55% of energy) is probably essential. In the subsequent months and years, the amount of energy required for growth decreases and in older child other components of energy expenditure, such as basal metabolic rate, thermoregulation and, above all, physical activity, become more important. The growth data of the STRIP trial support the safety of a low-saturated fat, low-cholesterol diet administered to infants aged > 7 months and continued from the first year of life throughout childhood, but the precise percentage of fat dietary intake supporting normal growth and development and maximally reducing atherosclerosis risk, is still unknown [43]. Therefore, the appropriate fat intake should be better recommended not a daily, but a several day-range basis.

### 2.3. Age-Modulated Nutrient Recommendations

The ‘programming’ hypothesis suggests that the future cardiovascular responses are established either in prenatal age or in response to early feeding exposures in life [33]. Human milk is universally recognized as the “gold standard” for infant nutrition so as every other nutritional strategy must be compared to it [47]. Breast milk contains an elevated amount of saturated fat and cholesterol but low amount of sodium. Infants who are breastfed have higher blood cholesterol levels at one year of age if compared to formula-fed infants, but when they become adults, these data are often inverted and adults who have been breastfed end to have lower blood cholesterol values. [48]. Furthermore, breast-feeding is associated with at least 2 behavioral benefits on cardiovascular risk: a better self-regulation on intake amounts, and a taste evolution that may improve acceptance and preference of healthy foods after weaning and later in life [49]. Evidence suggests that infants who were breastfed tend to have lower blood pressure values i later in childhood [50]. Breastfeeding seems to have a protective effect towards the development of future obesity, as reported in other systematic reviews [51]. The period from complementary feeding to the achievement of a mature diet, from 4–6 months to 2–3 years of age, represents a radical shift in pattern of food consumption. The introduction of solid food as a complement of breastmilk or formula milk should start at about 6 months of age to grant a sufficient amount of micronutrients, but the optimal methods to fulfill this goal are not clearly defined. [52]. Current feeding practices are influenced by small scale studies of infant feeding behavior [53], parental behavior, popular opinions and folk customs. New healthy foods may need to be introduced repeatedly to establish taste preferences [54]. Moreover, it is recognized that children ranging from 2 to 5 years of age are selective in their food choices. After 2 years of age, the amount of calories derived from fat should be gradually reduced to less than 30% of total daily calories. Calories no more introduced as fat should be replaced with grain products, fruits, vegetables, low fat milk products, legumes, lean meat, fish or other protein rich foods. In the age from 2 to 6 years, challenges are related to providing quality nutrient intake and avoiding excessive caloric intake. A significant amount of saturated fat and cholesterol derives from milk and dairy products in this age group, therefore a transition to low-fat milk and other dairy products is important. In the clinical practice, some parents and their children may over-interpret the need to restrict their fat intakes and for this reason it is important to give a higher accepted limit of fat intake; we must also emphasize that these recommendations are for average intake over several days, so that if fat-rich food are consumed, a compensation can be obtained eating healthier food in the other weekly meals. Since no single food item provides all the essential nutrients in the amounts needed, food variety seems to be a key stone in building an adequate diet. As children grow up, sources of food and influences on eating behavior increase; many meals and snacks are usually consumed away from home and often without supervision. Significant adverse changes occur in older children’s food consumption: they usually reduce or skip breakfast and increase intake of snacks, fried and nutrient-poor foods, sweetened beverages, as well as portion size at each meal. In addition, at this age dairy product consumption decreases and there is a shift away from fruit and vegetable consumption. This shift in dietary patterns in adolescence results in calcium, potassium, iron, zinc and vitamins intake below recommended levels, whereas sodium intake is far above recommended intake [55]. Adolescence is a nutritionally vulnerable developmental stage, as growth rate accelerates; therefore, counselling in late childhood and adolescence should be individualized for better dealing with contemporary lifestyles.

### 2.4. Dietary Recommendations for Children over the Past Twenty Years

In 1998 the American Academy of Pediatrics recommended on whether to promote dietary changes in all healthy children (population approach) or to identify and treat only children who are at the highest risk for the development of accelerated atherosclerosis in early adult life (individualized approach) [56]. In 2000 the Italian Society of Pediatric Nutrition (SINUPE) endorsed a similar document to identify and treat hypercholesterolemia in children in Italy, as shown in Table 1 [57].

Specific nutrient recommendations are as follows: no restriction of fat or cholesterol for infants <2 years when rapid growth and development require high energy intakes; nutrition adequacy should be obtained by eating a wide variety of foods; caloric intake should be adequate to support growth and development and to reach and maintain desirable body weight. In the population approach to lower cholesterol levels the Step-One diet is recommended: ≤30% and no less than 20% of calories from total fat; <10% of total calories from saturated fat; ≤10% of calories from polyunsaturated fat; cholesterol no more than 300 mg per day. This therapeutic diet should be prescribed in a medical setting with monitoring and follow-up provided by a health professional; if children do not reach the desirable cholesterol levels after 3–6 months of such diet, they require counselling to adopt the Step-Two Diet.

This regimen should start with accurate assessment of current eating patterns and instruction by a physician. It includes: no more than 30% and no less than 20% of calories from total fat; ≤7% of total calories from saturated fat; ≤10% of calories from polyunsaturated fat; no more than 200 mg/day of cholesterol. This dietary pattern requires careful planning to ensure adequacy of nutrients, vitamins and minerals and follow up by a qualified nutrition professional [56].

In 2005 the American Heart Association presented the Dietary Recommendations for Children and Adolescents endorsed by the American Academy of Pediatrics with new focuses on both caloric intake and eating behaviors [58]. In the Expert Panel on Integrated Guidelines for Cardiovascular Health and Risk Reduction in Children and Adolescents the first step proposed for management of children with identified lipid abnormalities was a focused intervention on diet and physical activity, with dietary accommodations that still follow the Step-One and the Step-Two Diet. The use of dietary adjuncts such as plant sterols or stanols was also proposed for children with primary hypercholesterolemia who do not achieve LDL-C goals with dietary treatment alone to avoid the necessity of drug treatment [6].

**Figure 1:**
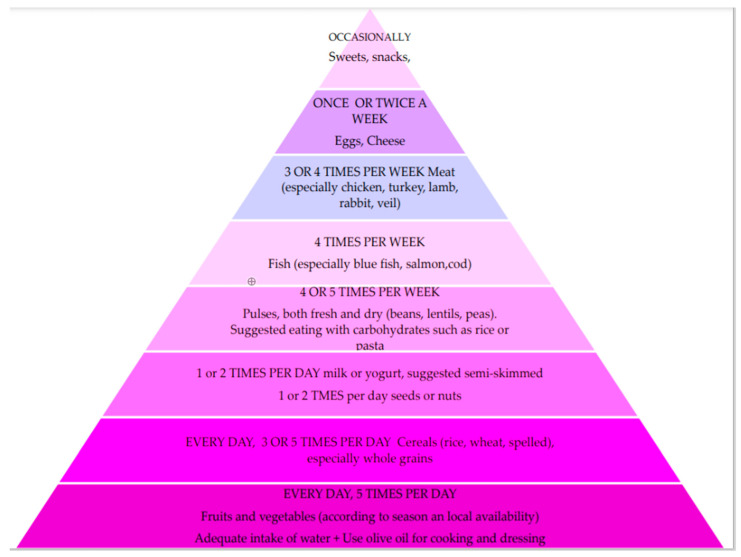
The Nutritional Diet Pyramid for children, modified from Giovannini et al. [57].

In the European Atherosclerosis Society (EAS) document for detection and treatment of FH in children and adolescents the Panel recommends a heart-healthy, fat modified diet (<30% calories from total fat, <7% of calories from saturated fat and <200 mg/day of cholesterol), including nutrient dense-foods with appropriate energy to maintain optimal body weight. Intake of fruit and vegetables, whole grains, low fat dairy products, legumes, fish and lean meats should be encouraged; the Mediterranean diet is referred to as an ideal model of heart-healthy diet (Figure 1).

**Figure 2:**
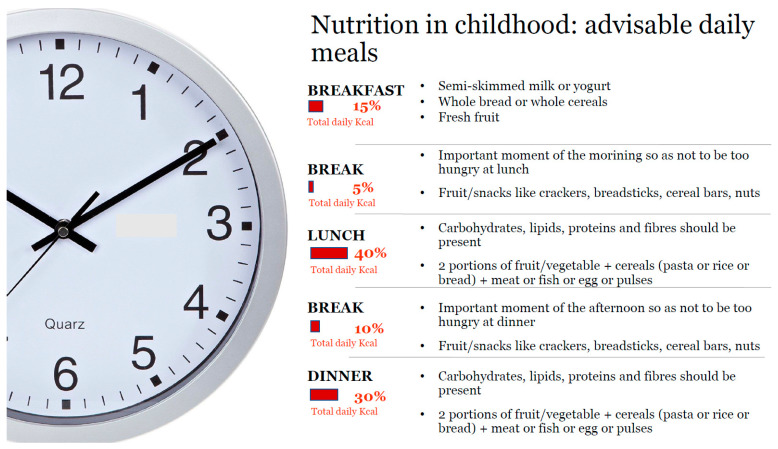
Advisable daily meal distributions in childhood, modified from Giovannini et al. [57].

The possibility of using functional foods is included; in this regard, foods containing added plant sterols/stanols, psyllium-enriched cereals, garlic extract, omega-3 fatty acids and soy proteins, have been evaluated in small studies in children with hypercholesterolemia. Anyway, no strong recommendations on functional foods utilization in childhood and adolescence are yet available. [11]. In the 2016 European Society of Cardiology (ESC) and EAS guidelines for the management of dyslipidemias, plant sterols/stanols are indicated for adults and children (>6 years) with FH [59]. In Italy, a heart-healthy diet should be in accordance with the Italian Society of Human Nutrition reference values (LARN) [55]. A daily recommended distributions of meals is shown in Figure 2. Recommended macronutrient intake for school-age children are shown in Table 2.

Daily energy intake must always be age related; protein intake 0.94–0.97 g/kg per day; carbohydrate and fat intake: 45–60% (sugar < 15%) and 20–35% (saturated fatty acids < 10%, polyunsaturated fatty acids 5–10%) of daily energy intake, respectively; fiber 8.4 g/1000 Kcal. General nutritional recommendations include increasing frequency intake of fruit, vegetables, legumes and fish, meantime decreasing meat consumption and introducing grain food, also according to the principles of the Mediterranean diet [60]. In the last years, the Nordic diet has emerged as a healthy eating option in Nordic countries (Denmark, Sweden, Finland) [61]; it is characterized by the intake of apples, pears and berries, root and cruciferous vegetables, cabbages, whole grain and rye bread as cereals, high intake of fish, low fat dairy products, potatoes and vegetable fats (margarine, vegetable oil). To our knowledge, the long-term effects of this diet on major chronic diseases have only been investigated in adults within Nordic counties [62,63]. Characteristics of Mediterranean Diet can be found in Table 3, comparison of the main feature of Mediterranean and Nordic diet can be found in Table 4.

## 3. Specific Dietary Interventions

### 3.1. Dietary Treatment for FH in Children

Early treatment of FH can reduce the negative impact of high LDL-C levels, improving endothelial function, reducing atherosclerosis progression and the risk of cardiovascular disease [11]. First-line treatment of FH is represented by diet and promotion of a healthy habits [6]. Patients and their families should undergo education targeting lifestyle management and should be informed about food choices. A certified pediatrician expert in nutrition should involve the whole family in laying the foundations of a healthy diet. A complete record of dietary habits must be obtained, and recommendations for a diet should be personalized and interpreted for each child to address individual diet patterns. The goal is to early establish correct habits that are most likely to be maintained over time, until adulthood [64,65]. The lipid intake, in particular cholesterol and saturated fats, is a major determinant of blood cholesterol levels. The 2015 EAS guidelines recommended a restriction of saturated fats intake <7% of total calories and daily cholesterol intake <200 mg. In children below 2 years of age, dietary restriction of fat and cholesterol is not recommended to prevent the risk of poor growth and developmental delay, due to their crucial role in brain and cognitive development. In school-age children challenges are related to achieving quality of nutrient intake, meantime avoiding excessive caloric intake. In children below two years of age, saturated fats and cholesterol mainly derive from dairy products, therefore it is important to promote consumption of low-fat milk and other dairy products [58]. The ideal diet should present the following characteristics: protein intake of 12–14% of total daily calories with an animal/vegetal protein ratio of 1:1; carbohydrates (mainly complex type) intake of 55–60% of total daily calories, lipid intake below 30% but not below 25% of total daily calories (saturated fats < 7%, monounsaturated 10–15% and polyunsaturated 5–10%). The goal should be a moderate fat intake, with primary sources of added fats coming from vegetable oils. The dietary scheme should consist of 5 daily meals: breakfast, morning break, lunch, afternoon break and dinner. Daily energy intake should be correctly provided as follows: 20% from breakfast and morning break, 40% from lunch, 10% from afternoon break and 30% at dinner. Weekly food frequency should be: meat 3 times/week (preferring lean meat), fish 4 times/week rich in DHA (blue fish, cod, salmon, tuna avoiding shellfish and clams), legumes 3–4 times/week, low-fat cheese 1–2 times/week, cold cuts 1–2 times/week and egg once/week [57]. The Mediterranean diet is the ideal model recommended, consisting of fruit and vegetables, grain products (especially whole grains), legumes, poultry and lean meats, fish and olive oil as the principal source of fats and low intake of salt. It encourages steam and oven cooking, limiting the use of fried foods [60,66]. A recent review has assessed the impact of diet on plasma lipids in patients with FH. A total of 19 RCTs (5 of which with pediatric population) encompassing 837 individuals with FH were included. In 10 out of 19 studies, a significant reduction in LDL-C was reported, including 8 dietary supplement interventions, 1 food-base intervention and 1 dietary counselling intervention. This systematic review first highlighted that the lack of effectiveness of diet in modulating LDL-C levels is likely due to biases in study designs rather than a true lack of effects. In fact, most RCTs with a low risk of bias were more likely to report significant reduction in LDL-C [67]. Diet and healthy lifestyle efficacy in children with FH is variable and it is modulated by genetic factors, such as Apolipoprotein E genotype (ApoE genotype E3/E4 is related to higher lipid profile but with a better response to diet intervention) [59]. Diet alone is often not able to achieve target levels for LDL-C and drug treatment is also required [58]. Physical activity should be promoted and sedentary lifestyle should be limited. It is strongly recommended the reduction of associated risk factors: cigarette smoke (also passive), obesity, diabetes and hypertension (11). Smoking habit should be discouraged firstly among parents and other family members, so as to reduce the risk for their children to adopt this habit [68].

### 3.2. Diet and Nutritional Intervention in Obesity

**Figure 3:**
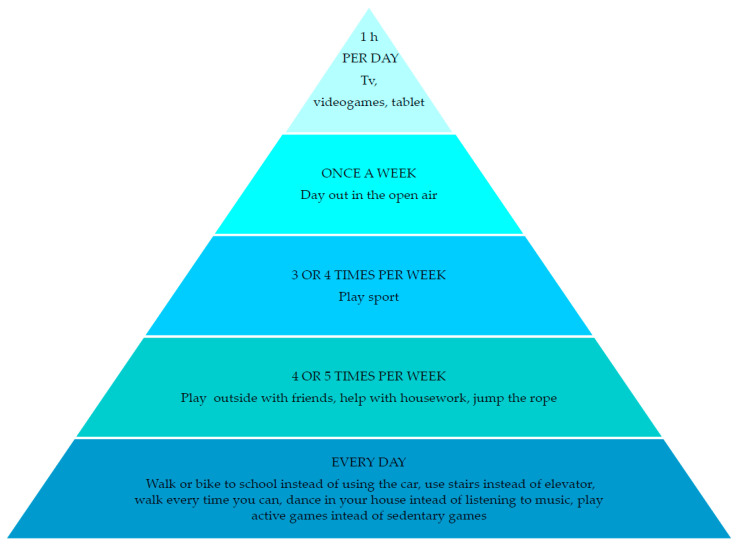
The physical activity pyramid for children, modified from Giovannini et al. [57].

Obesity is currently one of the most serious global public health problems and robust evidence demonstrates that physical, metabolic, cardiovascular and psychosocial complications are already present in obese children and worsen in adulthood. The WHO European Childhood Obesity Surveillance Program (COSI) revealed that overweight and obesity rates among primary-schoolchildren range from 15–52% in boys and from 13–43% in girls, with higher prevalence in Southern European Countries (2012) [16]. Healthy lifestyle is another milestone for cardiovascular health, as shown in Figure 3.

According to the Italian Government Surveillance Project ‘Okkio alla Salute’ between 2008 and 2019, in Italy the prevalence of obesity decreased from 12% to 9.4%, while overweight prevalence decreased from 23.2% to 20.4% in primary-school children [69]. This is the consequence of numerous interventions promoting healthy lifestyles, such as increasing physical activity and improving eating habits in the schools, implemented at regional and local levels. Obesity is frequently associated with multiple metabolic abnormalities that increase patient’s cardiovascular risk: dyslipidemia, insulin resistance and hypertension are the basic components of the metabolic syndrome. According to the International Diabetes Federation, the pediatric metabolic syndrome should be diagnosed in school-aged children (10 years old or more) when abdominal obesity is associated with two or more other clinical features as high blood pressure, elevated triglycerides levels, low HDL-C levels and increased glycemic levels [70]. Genetic, socio-economic and environmental factors play a role as metabolic syndrome drivers with unhealthy eating habits at the first place. A diet characterized by high protein content, saturated fats, refined grains, sugar and salty foods and a low consumption of fruits and vegetables, is recognized to worsen metabolic patterns associated with obesity and metabolic syndrome. Dyslipidemia, defined by one or more serum lipids out of range, represents one of the main comorbidities in pediatric obesity and its prevalence among overweight and obese children is about 46–50.4% [71,72]. In particular, in a study that included 139 children, ranging from 8 to 14 years of age, who were overweight or obese according to BMI z-score, detected dyslipidemia patterns were as follows: hypertriglyceridemia (31.9%), low HDL-C (29.7%), high non-HDL-C (15.8%), hypercholesterolemia (11.9%), high LDL-C (10.7%) [71]. BMI z-score showed a positive correlation with triglycerides (TG) and negative with HDL-C levels. The simultaneous presence of obesity and dyslipidemia (especially high TG levels) is associated with the risk of developing cardiovascular events in adulthood [73]. Specifically, TG/HDL-C ratio plays a decisive role and it can be used as a marker for identifying cardio-metabolic risk factors or signs of organ damage when >2.2 [74]. For this reason, it is recommended to screen overweight or obese children for dyslipidemia every three years since they reach six years of age; if they show a rapid increase in weight or develop other comorbidities, blood testing can be anticipated [6]. The main objective of the treatment of obese children and adolescents is a permanent change in their eating habits and lifestyle. A gradual reduction of BMI should be achieved through changes in diet and lifestyle, so as to obtain a negative caloric balance. As indicated by many expert panels, the whole family involvement and the setting of achievable goals are mandatory. Different authors reported that the prescription of a low caloric diet is not effective in the medium- and long-term management of obese children, being associated to relapses and failures, increased risk of dropout and progression into more complicated forms. The winning strategy starts from the analysis of the eating habits of the child and his family, for instance using a valid tool such as a food diary [75]. The main dietary advices summarized by the 2018 Consensus Position on Pediatric Obesity, issued by the Italian Society for Pediatric Endocrinology and Diabetology, are the following [76]: have an adequate breakfast, avoid eating between meals, have three meals and no more than two snacks per day, limit portions, avoid high-energy and low nutrient density foods (e.g., fruit juices or fast food), increase intake of fruit, vegetables and fiber-rich cereals. Nutritional recommendations for children with obesity can be found in Table 5.

If a low caloric diet should be prescribed, LARN reference values for Italian population have to be respected for different age groups. In selected patients with severe obesity a very low caloric diet may be prescribed for some months (no longer than 10 weeks) under close medical surveillance with the aim to induce rapid weight loss, followed by a less restrictive dietary regimen, balanced in macronutrients. The protein sparing modified fast is an example of very low caloric diet (600–800 Kcal per day, protein 1.5–2 g/kg of ideal weight, carbohydrates 20–25 g/day; multivitamins, minerals and water > 2000 mL per day). Reduced caloric intake (1000–1500 Kcal/day) is achieved through categories of food grouped by nutrient density, it is well accepted and produces a significant improvement of BMI in 8–12 years old children even in a long-term period. Replaced meals are not recommended, since efficacy and safety have not been tested in children and adolescents; hypocaloric diets with low glycemic load, although having an effect on satiety, have not superior effect compared with other dietary approaches in the medium term [77]. The diet effect on these children is evaluated using the BMI-SDS (Standard Deviation Score); a reduction >0.5 in a growing child correlates with better body composition and decreased CHD risk [78]; waist circumference and skinfold thicknesses can be also used to measure fat percentage, but they do not offer other benefits with regard to BMI [79]. Several studies have shown that the discontinuation of weight management programs can discourage families, hinder the action of clinicians and result in inefficient use of clinical resources. Attendance rates and patients’ enrollment present similar challenges, which must be addressed in order to optimize the strategy of care. Programs not meeting families’ needs and logistical barriers are reported as the most commonly causes of attrition and failure in the management of pediatric obesity [80,81,82]. Several systematic reviews and meta-analyses on prevention and treatment of overweight and obese children and adolescents indicate that weight control may be obtained by multicomponent intervention focused on life-long changes of dietary habits and lifestyle, involving the whole family, the school and the communities. The effectiveness of these treatment programs on weight reduction on the long term from childhood to adulthood is still unknown and it needs further long-term studies.

### 3.3. Dietary Complements

In recent years, there is an increasing interest on functional foods and nutraceuticals. These products may act as a support therapy for lowering plasma cholesterol, LDL-C and triglycerides, especially in primary prevention of CHD in hypercholesterolemic subjects, whose blood cholesterol level is not within normal range but not high enough to require pharmacological treatment [83]. In 2011, the Expert Panel on Integrated Guidelines for Cardiovascular Health and Risk Reduction in Children and Adolescents included for the first time the use of dietary adjuncts such plant sterols or stanols for children with primary hypercholesterolemia who do not achieve LDL-C goals with dietary treatment alone to avoid the necessity of drug treatment [6]. According to current evidence, nutraceuticals could exert significant lipid-lowering activity and their introduction in the dietary intervention has many advantages. Multiple mechanisms, such as inhibition of the absorption, synthesis and regulation of cholesterol metabolism are involved [84]. Nutraceuticals can act simultaneously on multiple stages of lipid-induced vascular damage; therefore, they can improve the lipid-lowering effects when used in combination with diet, pharmacological treatment or other nutraceuticals. In addition, more, they can have a variety of positive pleiotropic effects, including improvement of arterial stiffness and endothelial dysfunction, as well as anti-inflammatory and anti-oxidative properties. Most of them originate from vegetable products. These compounds have a moderate lipid-lowering action and their use has proved to be safe and frequently well tolerated. Nutraceuticals do not represent an alternative, but a complement to the dietary-nutritional intervention that is the first line approach [85]. The experience of using nutraceuticals in pediatric age is still limited; most of the studies analyze the integration with alimentary fibers or with phytosterols/stanols [86]. Lipid-lowering effect of the fibers is associated to inhibition of the cholesterol absorption. Its intake should mainly derive from a correct intake of fruit and vegetables. In case of persistence of elevated levels of total cholesterol and LDL-C, supplementation with soluble fiber can improve the lipid profile, without significant adverse health effects [87]. Plant sterols are naturally occurring compounds found in plant cell membranes that are structurally similar to cholesterol. They compete with it and inhibit the absorption of cholesterol in the small intestine, resulting in lower plasma LDL-C levels. Sterols can be used in children with mild hypercholesterolemia, but there are no long-term safety data [88]. N-3 polyunsaturated fatty acids (PUFAs) with a double bond in position 3 at the end of the carbon chain, are found in animal (e.g., fish, egg, squid) and plant (e.g., algae, walnut, seed) sources. In recent years, the European Food Safety Authority (EFSA) and the American Heart Association (AHA) have recognized n-3 PUFAs as preventive nutraceuticals for CHD. EFSA established a claim in 2010 indicating that the intake of at least 2 g/day of docosahexaenoic acid (DHA) and eicosapentaenoic acid (EPA) has the ability to maintain normal blood TG levels. AHA indicated doses from 2 to 4 g/day of EPA/DHA to reduce TG levels by 25–30%. All these guidelines agree about the high safety of PUFAs [89]. According with data available in the literature and the European Guidelines (EAS 2016) we can suggest the use of functional foods based on fiber and plant sterols in the treatment of children with genetic-familial dyslipidemia from 6 years of age upwards.

## 4. Conclusions

It is worldwide recognized that cardiovascular diseases are related to dyslipidemia, hypertension, obesity, diabetes mellitus, tobacco use and physical inactivity and the principal causes of these risk factors are adverse behaviors and lifestyles. In the last 20 years, an increasing attention has been given to the importance of nutrition in the first years of life, also including fetal period, as primordial prevention. A prudent diet is central to prevent the development of dangerous serum lipid levels, excessive adiposity and elevated blood pressure. Prevention of the CHD through intervention in childhood is supported by the fact that dietary habits and food preferences are formed early in life and that lifestyle and eating habits set in the family environment since childhood tend to be maintained over time throughout the life span. Nowadays, promoting lifestyle modifications and the pursuit of psychological well-being are universally recognized as major factors that may provide more enduring effects, as well as the reduction of the so-called allostatic load in pediatric patients and their families, thus improving the approach to the treatment of children and adolescents at high cardiovascular risk [90].

## Figures and Tables

**Table 1:** Dietary recommendations for children with hypercholesterolemia, modified from Giovannini M. et al. [57].

DIETARY RECOMMENDATIONS FOR CHILDREN
GENERAL (population approach)
No restriction of fat or cholesterol intake for infants of age < 2 years (rapid growth) Caloric intake should be adequate to support growth and achieve desirable body weight Nutritional adequacy should be achieved by eating a wide variety of foods
LOW-FAT DIET (for hypercholesterolemia)
STEP ONE DIET
Total fat ≤ 30% (no less than 20%) of total daily calories Saturated fats < 10% of total daily calories Polyunsaturated fats ≤ 10% of total daily calories Cholesterol ≤ 300 mg/day
STEP TWO DIET
Total fat ≤ 30% (no less of 20%) of total daily calories Saturated fats ≤ 7% of total daily calories Polyunsaturated fats ≤ 10% of total daily calories Cholesterol ≤ 200 mg/day

**Table 2:** Recommended macronutrients intake for school-age children, modified from SINU [55].

Adequate energy intake depending on age and sex (1372–2499 Kcal/day) Proteins 12–14% of total daily energy (0.94–0.97 g/kg/day) Carbohydrates: 55–60% of total daily energy (sugar <15%) Fats: 20–35% of total daily energy (saturated-fat <10%, polyunsaturated fats 5–10% of total energy)

**Table 3:** Main characteristics of Mediterranean Diet, derived from Widmer Rj et al. [60].

Ideal model of diet even in “non-Mediterranean” countries High intake of fresh fruits and vegetables Daily intake of cereals (such as pasta, bread and others) High intake of blue fish Low intake of meat, preferring sheep, goat, chicken or turkey Daily use of olive oil, rich in unsaturated fatty acids High fibre intake Moderate amounts of food for meal Fresh and seasonal food Importance of conviviality

**Table 4:** Comparison of some aspects of Mediterranean and Nordic diet, derived from Galbete C. [63].

HEALTHY DIET OPTIONS IN EUROPE: NORDIC DIET AND MEDITERRANEAN DIET	
NORDIC DIET	MEDITERRANEAN DIET
Whole grain/rye bread Berries Apple and pear Fish Dairy products Root vegetables Cabbage/cruciferous vegetables Vegetable fats Potatoes	Cereals Fruits/Nuts Vegetables Fish Dairy products Meat Legumes and pulses Olive oil Alcohol

**Table 5:** Nutritional recommendations for obese children, derived from Valerio G et al. [76].

Main Dietary Advices for Obese Children
Consume adequate breakfast Avoid nibbling/avoid eating between meals Have three main meals and no more than two snacks per day Avoid high-energy food Limit portions Increase intake of fresh fruit and vegetables Increase intake of fiber-rich cereals

## Data Availability

Not applicable.

## Abbreviations

AHA	American Heart Association
BMI	Body Mass Index
CHD	Coronary Heart Disease
COSI	European Childhood Obesity Surveillance Program
DHA	Docosaexahenoic Acid
DISC	Dietary Intervention Study in Children
EAS	European Atherosclerosis Society
EFSA	European Food Safety Authority
EPA	Eicosapentaenoic acid
FH	Familial Hypercholesterolemia
LARN	Italian Society of Human Nutrition reference values
LDL-C	Low Density Lipoprotein Cholesterol
HDL-C	High Density Lipoprotein Cholesterol
HoFH	Homozygous Familial Hypercholesterolemia
PUFAs	N-3 polyunsaturated fatty acids
SINUPE	Italian Society of Pediatric Nutrition
STRIP	Special Turku Coronary Risk Factor Intervention Project for Babies
TG	Triglycerides
